# Econometric estimation of WHO-CHOICE country-specific costs for inpatient and outpatient health service delivery

**DOI:** 10.1186/s12962-018-0095-x

**Published:** 2018-03-19

**Authors:** Karin Stenberg, Jeremy A. Lauer, Georgios Gkountouras, Christopher Fitzpatrick, Anderson Stanciole

**Affiliations:** 1 Department of Health Systems Governance and Financing, World Health Organization, Geneva, Switzerland; 2 School of Pharmacy, University of Nottingham, Nottingham, UK; 3 Department of Neglected Tropical Diseases, World Health Organization, Geneva, Switzerland; 4 United Nations Population Fund, Asia and Pacific Regional Office, Bangkok, Thailand

**Keywords:** Cost, Regression analysis, Estimates, Inpatient, Outpatient

## Abstract

**Background:**

Policy makers require information on costs related to inpatient and outpatient health services to inform resource allocation decisions.

**Methods:**

Country data sets were gathered in 2008–2010 through literature reviews, website searches and a public call for cost data. Multivariate regression analysis was used to explore the determinants of variability in unit costs using data from 30 countries. Two models were designed, with the inpatient and outpatient models drawing upon 3407 and 9028 observations respectively. Cost estimates are produced at country and regional level, with 95% confidence intervals.

**Results:**

Inpatient costs across 30 countries are significantly associated with the type of hospital, ownership, as well as bed occupancy rate, average length of stay, and total number of inpatient admissions. Changes in outpatient costs are significantly associated with location, facility ownership and the level of care, as well as to the number of outpatient visits and visits per provider per day.

**Conclusions:**

These updated WHO-CHOICE service delivery unit costs are statistically robust and may be used by analysts as inputs for economic analysis. The models can predict country-specific unit costs at different capacity levels and in different settings.

**Electronic supplementary material:**

The online version of this article (10.1186/s12962-018-0095-x) contains supplementary material, which is available to authorized users.

## Background

Health planners concerned with evidence-informed decision making and resource allocation rely on high quality information regarding the resources needed to implement investment strategies, and comparing these against current and future budgetary constraints. Information on costs is essential to inform discussions around value for money and efficiency. Unfortunately cost data is sparse in many settings, especially in low- and middle income countries, where ambitious health targets are now being set for the 2030 Sustainable Development Goals (SDGs). Challenges include insufficient allocation and/or inefficient allocation of resources towards health priorities [1]. Having access to accurate and reliable information on the cost of health services can serve various purposes including discussions on affordability and financial sustainability, budgeting, cost-effectiveness and cost–benefit analysis [2].

Literature related to estimates on the so-called ‘unit costs’ of specific health interventions is a growing field, yet the transferability of such findings from one setting to another is limited [2, 3]. For example, the Access, Bottlenecks, Costs and Equity (ABCE) initiative from the University of Washington’s Institute for Health Metrics and Evaluation has been collecting primary data on health care facility costs. However, the focus has been limited to Ghana, Kenya, Uganda and Zambia and there is no intention to create generalized global estimates [4–7]. Similarly, the recently established Global Health Cost Consortium at the University of Washington (https://ghcosting.org/) intends to produce unit cost estimates that can be adapted to local settings, but its scope is limited to TB and HIV services, and will not provide cost estimates for general inpatient or outpatient care.

To our knowledge, the WHO-CHOICE (CHOosing Interventions that are Cost Effective) project is the only programme seeking to collect and standardise estimates of the costs related to facility-based service delivery, to compare these across countries, and to provide country-specific estimates of facility service costs as a global public good. WHO-CHOICE estimates are produced for all countries and can therefore be used in settings where no local data is available. Estimates are based on modeling of primary and secondary data, and are derived from a model that provides the best fit according to global data. Such models will not yield perfect predictions for all countries, and it is therefore maintained that whenever good quality country unit cost data is available from a representative sample, this should be used rather than using the CHOICE predictions.

Under WHO-CHOICE, WHO has collated facility cost data from countries since 2000. These data have served to inform estimates for country-specific costs related to health service utilization—with estimates for cost per inpatient day and outpatient visit. The service delivery unit cost estimates are used to support cost-effectiveness analysis within the WHO-CHOICE project, among other things.

- *Inpatient day* The estimated cost of a hospital bed-day reflects only the “hotel” component of the hospital cost—i.e. it excludes the cost of drugs and diagnostic tests but includes costs such as personnel, capital infrastructure and equipment, laboratory, maintenance and other operational costs of the hospital, as well as food costs. The intent is to produce a measure that covers those components that are assumed to be standardised across different diseases and treatments.
- *Outpatient visit* Similarly, WHO-CHOICE outpatient costs include components not specific to the disease or treatment, but those largely assumed to be standardised across disease conditions for which the care is provided: namely personnel, capital infrastructure and equipment, laboratory, maintenance and other operational costs of the health facility. In recognition of the fact that costs for equipment, maintenance etc., may vary depending on the setting in which care is provided, inpatient and outpatient care costs are estimated for different types of providers and levels of the health system.

The use of standardised estimates for the service delivery component of intervention costs ensures that interventions can be compared fairly, using consistent price assumptions. Inputs into the service production process (including technology, prices and production efficiency) change over time, and WHO-CHOICE seeks to regularly provide updated estimates. WHO-CHOICE estimates are particularly useful for low- and middle income countries that may not have such data readily available.

In this paper we describe a process whereby country data sets were gathered and multivariate regression analysis was used to explore the determinants of variability in unit costs, in order to produce updated WHO-CHOICE estimates.

The previous round of WHO-CHOICE results for facility cost are henceforth referred to as “the first round analysis” [3, 8, 9]. Estimates are publicly available (http://www.who.int/choice) and have been widely used by researchers, academics and analysts at both global and country level [10, 11]—for example publications see http://who.int/choice/documents/en/).

## Methods

The “Methods” section first describes the data collection process followed by the econometric analysis.

### Data collection

As a first step a literature review was undertaken with the aim of identifying variables and methods that should be taken into account during data collection and analysis [12].

Experience from prior work suggested that on the ground ‘bottom-up’ facility-level estimation is not a cost-effective approach for producing large data sets, given the large costs involved with primary data collection. Instead we made use of existing data for secondary analysis. Cost data were gathered 2009–2010 through three mechanisms:

- Authors identified through the literature review were directly contacted;
- A public call went out for cost data; and
- Websites of public institutions were searched for publicly available data.

The majority of data was gathered from respondents to the public call. Most of the sources identified through the literature review did not report facility-specific unit costs estimates, but instead presented only the sample average. Therefore, data from only two studies identified though the literature review were ultimately included in the estimation dataset (Additional file 1: Annex S1). In-depth examination of available online public data bases revealed limited usefulness of such data as they generally lacked several of the variables required to inform the analysis. Only one database found on-line was considered useful for extracting data [13].

The public call for cost data was released and widely disseminated on leading academic and international development websites in early 2009. The public call asked respondents to provide datasets of unit costs from at least 30 health facilities at any level (primary, secondary, tertiary or a mix). The data request covered a range of indicators, including: average unit costs (per bed day, admission and outpatient visit); the breakdown of each unit cost estimate by input category (salary, drugs, other supplies, capital), the proportion of recurrent to total costs, the proportion of drugs to recurrent costs, the proportion of ancillary costs to recurrent costs; average and recurrent unit costs of laboratory tests and diagnostic procedures; various determinants of costs and efficiency (e.g. average length of stay, occupancy rate, bed turn over, number of medical staff per bed, number of outpatient visits per medical staff per day, number of beds); and utilization data (e.g. number of bed days, number of admissions, number of outpatient visits and number of ancillary services by type of service).

Respondents to the public call were sent a scoping questionnaire to assess the type of information available. Out of 60 proposals, and considering geographic and income-level representation, a total of 30 respondents were sent the final data collection form of which 27 provided final data sets and were remunerated. Most respondents worked at public institutions and had access to data on resource use per facility, which had originally been collected to inform provider payment schemes and/or to monitor health system performance.

A standard template was used for extracting data. The range of variables collected drew upon earlier work, [3, 8, 9] and included facility size, level of care (Box 1), public/private affiliation (Box 1), number of available beds, number of outpatient visits, number of staff (by category), reference year for cost data, and a breakdown of costs into various components including medicines, salaries, laboratory and soforth; as well as the estimated split in costs between outpatient care and inpatient care, if available (see Additional file 1: Annex S2 for a complete list of variables).

Data was collected with a specific intention to assess capacity utilization as an explanatory variable [3]. Higher capacity utilization should result in lower predicted unit cost, as fixed costs are spread across a greater number of outputs. For inpatient care we collected data on the percentage of beds occupied, while for outpatient care, we collected data on total number of patient visits and total number of staff, in order to calculate the number of visits per provider per day as a capacity measure.

Respondents’ files were screened for data quality and consistency with the requested data format. Unit costs were extracted from the data files. Quality control mechanisms included recalculation of the service delivery inpatient and outpatient cost in accordance with the research protocol and instructions sent to data providers. Data cleaning comprised consistency checks and when needed was followed by discussions with data suppliers to ensure that the data submitted corresponded to standard definitions and requested specification. Some of the missing variables were directly derived, when possible, from other variables from the same observation point (e.g. occupancy rate calculated from number of beds and number of bed-days).

Data was gathered for a total of 30 countries. Sample size ranged from 9 to 6725 (median 36) observations for health centres per country-specific dataset, and from 1 to 4938 observations for hospitals (median 42, see Additional file 1: Annex S1 for details). One observation represents one facility in a given year.

Data collection and cleaning resulted in a dataset that was significantly (six times) larger compared to the previous WHO-CHOICE facility cost database. The majority of new cost data referred to year 2007. Costs collected in local currency units were converted to 2007 International dollars by means of Gross Domestic Product (GDP) deflators [14] and purchasing-power-parity exchange rates.

### Box 1 Definition of facility level and ownership/affiliation

#### Five levels of care were considered

1. Health centres with outpatient services only (no beds)
2. Health centres with beds
3. Primary-level hospital: Hospitals intended primarily for treating simple cases (e.g. “district hospital”)
4. Secondary-level hospital: Hospitals intended primarily for treating referral cases (e.g. “specialist hospital”)
5. Teaching hospital: Hospitals intended primarily for treating referral cases, with a teaching component (e.g. “teaching hospital”).

For further information on definition of hospital levels, reference is made to Barnum and Kutzin [15].

- Primary-level hospital: Have few specialities, mainly internal medicine, obstetrics-gynecology, paediatrics, general surgery or just general practitioners; limited laboratory services are available for general but not for specialized pathological analysis; bed size ranging from 30 to 200 beds; often referred to as district hospitals or first level referral.
- Secondary-level hospital: Highly differentiated by function with five to ten clinical specialities; bed size ranging from 200 to 800 beds; often referred to as provincial hospital.
- Tertiary-level hospital: Highly specialized staff and technical equipment, e.g. cardiology, ICU and specialized imaging units; clinical services are highly differentiated by function; might have teaching activities; bed size ranging from 300 to 1500 beds; often referred to as central, regional or tertiary level hospital.

#### Three categories for affiliation were considered

- Public
- Private
- Not for profit private providers (i.e. faith-based, mission, or non-governmental organization).

### Econometric analysis

STATA software was used for data analysis [16]. Comparison with data previously collected (i.e. for the first round analysis) revealed that the new data had very different characteristics. Some of these differences were due to different variables having been collected, as a result of further development of data collection methods and instruments. Moreover, there was strong evidence of statistical heterogeneity in those variables that could be directly compared. As a result the old and the new datasets were not pooled, and regression analysis was performed using only the new dataset. Findings from the original literature review were also excluded unless authors had responded to the invitation to make available their datasets for inclusion in the analysis.

We adopted an approach derived from the economic literature on ‘hybrid cost functions’ [17, 18].

A log-cost function faced by a health facility is assumed to depend on:

- A log-additive vector of input prices, and
- An unknown function of:
  - A set of output indicators, and
  - A set of variables indicating the facility type.

Drawing upon previous work, various logarithmic models were tried and tested [3, 8, 9]. Variables were chosen based on the following criteria:

- The variable is a known determinant of unit cost,
- Measurement data for the variable are readily available,
- The variable performs well in regression models.

Previous experience suggested that some variables are more capable of influencing the outcome of the analysis than others [3, 8, 9]. For instance, the level of the health facility (i.e. primary, secondary or tertiary) is a main determinant of cost. Other variables, such as the proportion of emergency admissions, did not prove to be as important. Experimentation with different variables showed that a restricted list was preferable.

Unfortunately several country datasets had key variables missing, in particular regarding the breakdown of costs into components (e.g. salaries, drugs, lab tests and other costs), which affected the variables that could be used for the regressions. We explored methods for imputing data, but were unable to find a model specification with imputed values that performed sensibly in either the regressions or with respect to simple descriptive and summary statistics. Observations with missing essential data were therefore dropped. All 30 data sets were included but observations used in the final analysis were reduced from a total of 19,008—3407 (outpatient) and 9028 (inpatient)—see Additional file 1: Annex S1.

#### Model specification: inpatient care

The relationship between the outpatient/inpatient unit cost and explanatory variables was explored using multiple regression analysis—Ordinary Least Squares (OLS). The dependent variables and the continuous explanatory variables were transformed into natural logarithms. This has the advantage of coefficients being readily interpreted as elasticities. Country dummies were included in the models to address the impact of large data sets from Brazil and Colombia.

For the inpatient unit cost model the dependent variable is one bed-day. The functional specification may be written as:

$$\ln IUC_{i} = a_{0} + a_{i} \cdot\mathop \sum \limits_{i = 1}^{n} \ln X_{i } + e_{i} , \quad i = 1 \ldots n$$

where ln IUCi is the natural log (ln) of cost per inpatient day in 2007 US$ in the ith facility; α0 and α1…n are the estimated parameters; the *Xi* are the explanatory variables transformed into natural logarithms for continuous variables; and *e* represents the error term.

Table 1 lists the explanatory variables included in the final regression.

**Table 1:** Descriptive statistics of sample used for final inpatient care estimates (N = 3407)

	Description	Mean	SE
Ln cost per bed day	Natural log of cost per bed day in 2007 I$	181.59	241.21
Ln GDP per capita	Natural log of GDP per capita in 2007 I$	8788.86	3729.88
Ln occupancy rate	Natural log of bed occupancy rate	0.23	0.33
Ln ALOS	Natural log of Average Length of Stay (ALOS)	4.16	2.33
Ln admissions	Natural log of total inpatient admissions	4700.00	7416.81
District	Dummy variable for level 3 facilities^a^ (equivalent to district hospitals)	0.80	0.40
Teaching	Dummy variable for level 5 facilities (teaching hospitals)^a^	0.09	0
Public	Dummy variable for public hospitals^b^	0.58	1
Private	Dummy variable for private hospitals^b^	0.16	0
Brazil	Dummy variable for Brazil. Brazil = 1	0.58	1

^a^Dummies for hospital level are compared with level 4 facilities

^b^Dummies for hospital ownership are compared with private not-for profit hospitals

#### Model specification: outpatient care

For the outpatient unit cost model the dependent variable is one outpatient visit. The best performing approach for outpatient visits examined a pooled sample of health centers and hospitals.

The algebraic form of the outpatient unit cost model is:

$$\ln OUC_{i} = a_{0} + a_{i} \cdot\mathop \sum \limits_{i = 1}^{n} \ln X_{i } + e_{i} , \quad i = 1 \ldots n$$

where ln OUCi is the natural log (ln) of cost per outpatient visit in 2007 I$ in the ith facility; a0 and a1 are the estimated parameters; the *Xi* are the explanatory variables; and *e* represents the error term.

Table 2 lists the explanatory variables included in the final outpatient care regression. This includes an additional dummy specifically for Brazilian level 3 facilities, given that data from Brazil constituted a significant share of the sample for this level of hospitals.

**Table 2:** Descriptive statistics of sample used for final outpatient care estimates (N = 9028)

	Description	Mean	SE
Ln cost per visit	Natural log of cost per outpatient visit in 2007 I$	15.56	44.02
Ln GDP per capita	Natural log of GDP per capita in 2007 I$	7947.64	4618.50
Ln visits	Natural log of outpatient visits per facility	62,924.53	118,929.70
Ln visits per provider	Natural log of visits per provider per day (nurses, general practitioners)	5.71	10.90
Urban	Dummy variable for urban facility location^a^	0.21	0.41
Public	Dummy variable for public hospitals^b^	0.75	0.44
Private	Dummy variable for private hospitals^b^	0.12	0.32
Level 2	Dummy variable for level 2 facilities^c^	0.15	0.35
Level 3	Dummy variable for level 3 facilities^c^	0.25	0.43
Level 4	Dummy variable for level 4 facilities^c^	0.08	0.26
Colombia	Dummy variable for Colombia. Colombia = 1	0.10	0.30
Brazil	Dummy variable for Brazil. Brazil = 1	0.62	0.49
Brazil level 3	Dummy variable for Brazil level 3 facilities	0.18	0.39

^a^ Dummy for urban location is compared with rural location

^b^Dummies for facility ownership are compared with private not-for profit facilities

^c^Dummies for level of care are compared with level 1 facilities. Level 5 facilities are assumed to have the same outpatient costs as level 4 facilities

#### Model-fit

The tests used for judging model validity and the goodness of fit included the Breusch–Pagan/Cook–Weisberg test for heteroskedasticity [19], Ramsey’s regression specification-error test for omitted variables, the tolerance test and its reciprocal variance inflation factor, plots of the residuals versus the fitted values, plots of the residuals versus the independent variables, plots of the predicted values versus the continuous independent variables, estimates of adjusted R-squared, the Akaike information criterion, the Bayesian information criterion, and F-statistics of the regression model.

Robust estimation methods were used (i.e. the *Stata* command “robust”), in order to control for the effect on the estimate of standard errors caused by ‘clustering’ (i.e. the inclusion of multiple observations per country).

#### Predicted values and uncertainty analysis

WHO-CHOICE draws upon the prediction models described above to derive cost estimates for all member states. For the prediction of unit costs for inpatient days and outpatient visits, we use country-specific values where possible (i.e. GDP, country-specific dummy variables). Other explanatory variables, particularly those related to capacity utilization (e.g. occupancy rate, average length of stay), rely on representative ‘average values’ which can be set to normative values, sample medians, or to other values as appropriate. These ‘average values’ have desirable properties that may not always be possessed by the sample observations for that country [3]. Lacking appropriate norms, we applied the 80th percentile (p80) values from the sample of facilities used for the final regression models (Table 3). This is consistent with previous rounds of WHO-CHOICE unit cost estimates for which a 80% capacity level has been assumed.

**Table 3:** Values of variables used for prediction of the unit cost

Inpatient care	Proxy for size	Proxy for capacity	Proxy for intensity of care provided
	Total inpatient admissions per facility per year (p80)	Bed occupancy rate (p80)	Average length of stay, inpatient (p80)
Facility level 3	4971	0.756	7.14
Facility level 4 and 5	14,028	0.810	9.75

Outpatient care	Total outpatient visits per facility per year (p80)	Visits per provider per day (p80)
Facility level 1	67,656	8.96
Facility level 2	46,434	9.52
Facility level 3	93,739	3.22
Facility level 4 and 5^a^	281,156	2.36
Additional variables		
Ownership is set to public provider; location is set to urban location for outpatient care		

For definition of levels of care see Box 1

^a^Outpatient visit costs at level 5 are assumed to be the same as those for level 4

As a first step, regression results (coefficients and variance–covariance matrix) were used to predict country-specific unit costs within a 95% uncertainty interval (UI). For the generic set of WHO-CHOICE estimates we assume public provision at all facility levels (however the model can also be set to predict private provider costs). The initial analysis based variables (GDP I$ per capita) on year 2007, since the majority of data in our dataset (92%) referred to this year. We have since applied the same model in order to derive estimates for 2010, which is a year consistent with the Global Burden of Disease Study (GBD) 2010 data [20], and thus useful for cost-effectiveness analysis drawing upon the GBD 2010 estimates. Explanatory variables were based on data for 2010 (i.e. GDP per capita in 2010 I$) and other values described in Table 3.

Next, we undertook adjustments to ensure that drug costs were not included. Data providers had reported challenges relating to apportioning drug costs between inpatient and outpatient care. This was handled by applying a proportional adjustment ratio, derived from previous work on inpatient care [8], to the reported cost estimates, thus adjusting these downwards in order to capture only the general, standardized components of the facility visit and bed-day, as described above. We set drug dummy variables to 0 and 1 respectively in order to estimate the average estimated contribution of drug costs across countries (47.5%) in previous work [8]. A similar approach can be adopted for separating out food costs from the reported inpatient costs (9.9%) in the first round analysis. Due to lack of data regarding adjustment ratios for outpatient care related drug costs, we applied the ratio derived from previous inpatient care models to outpatient visits as well. The adjustment ratios were uniformly applied to the regression results across all country estimates.

As described in previous analysis, [8] the re-transformation of predicted log unit costs (iuc_i_) gives the median and not the mean of the distribution. One widely-used solution to this retransformation problem is Duan’s ‘smearing’ method [21]). The method is non-parametric, because it does not require that the regression error have any specific distribution. A smearing factor can be estimated following three steps: (i) Estimation of regression residuals, r_i_. (ii) Exponentiation of regression residuals to the power *e*, exp(r_i_). (iii) Averaging of the exponentiated residuals 1/n *∑exp(r_i_). The smearing factor is then multiplied by the re-transformed log unit costs (i.e. exp(c_i_)). This is the method applied in the Excel-based WHO CHOICE tools, which are available for countries to make their own predictions.

Unfortunately, Duan’s method requires that the distribution of the errors be homoscedastic. To test the sensitivity of our best estimates to this assumption and to construct 95% UIs, we employed a Bayesian approach. For each log prediction, we used the mean and standard error to draw 1000 random values, exponentiated each of these 1000 values and then extracted the mean, standard deviation and 2.5th and 97.5th centile values. For outpatient care, the two retransformation methods produced unit cost estimates that were equivalent on average. For inpatient care, the Bayesian approach produced unit cost estimates that were 4% higher on average. Given that the 95% UIs overlapped with estimates produced by Duan’s method, we present country-specific estimates obtained using the Bayesian approach.

## Results

### Descriptive statistics

Tables 1 and 2 show the variable names, description, mean and standard error for the data sets on inpatient and outpatient care respectively.

### Explanatory power

Tables 4 and 5 show the final regression models for inpatient and outpatient unit costs with 95% confidence intervals. All variables are significant, with most variables highly significant with p < 0.001. The inpatient cost model is slightly better performing with an adjusted R squared of 0.760 compared to 0.658 for the outpatient care model.

**Table 4:** Regression coefficients and 95% confidence interval: natural log of cost per inpatient bed day expressed in 2007 I $

	Regression coefficient	95% confidence interval
Ln GDP per capita	1.192***	[1.111, 1.272]
Ln occupancy rate	− 0.0201**	[− 0.0340, − 0.00623]
Ln ALOS	− 0.600***	[− 0.649, − 0.550]
Ln admissions	0.0252*	[0.00471, 0.0457]
District	− 0.204***	[− 0.275, − 0.132]
Teaching	0.257***	[0.163, 0.351]
Public	− 0.144***	[− 0.182, − 0.107]
Private	0.110***	[0.0710, 0.148]
Brazil	− 1.638***	[− 1.694, − 1.583]
Constant	− 4.277***	[− 5.035, − 3.519]
Observations	3407	
*R* ^2^	0.760	
Adjusted *R*^2^	0.760	
F_stat	1070.3	
Duan’s correction factor	1.054	
Variance inflation factor	1.721	

Note only facilities of types 3, 4 and 5 are included in this regression model

* *p* < 0.05, ** *p* < 0.01, *** *p* < 0.001

**Table 5:** Regression coefficients and 95% confidence interval: natural log of cost per outpatient visit expressed in 2007 I $

	Coefficient	95% confidence interval
Ln GDP per capita	0.865***	[0.826, 0.905]
Ln visits	− 0.0142*	[− 0.0272, − 0.00119]
Ln visits per provider	− 0.0412***	[− 0.0578, − 0.0246]
Urban	0.352***	[0.268, 0.435]
Public	− 0.290***	[− 0.330, − 0.249]
Private	0.0532*	[0.00479, 0.102]
Level 2	0.208***	[0.144, 0.271]
Level 3	0.304***	[0.213, 0.395]
Level 4	0.348***	[0.279, 0.417]
Colombia	0.628***	[0.542, 0.713]
Brazil	− 1.563***	[− 1.656, − 1.470]
Brazil level 3	− 0.245***	[− 0.337, − 0.153]
Constant	− 4.534***	[− 4.797, − 4.271]
Observations	9028	
*R* ^2^	0.658	
Adjusted *R*^2^	0.658	
F_stat	1635.7	
Duan’s correction factor	1.271	
Variance inflation factor	3.209	

* *p* < 0.05, ** *p* < 0.01, *** *p* < 0.001

Signs of the coefficients are consistent with expectations in both models, with more detailed interpretation below. In both models, GDP per capita is a highly significant proxy for price level but also for the level of technology.

### Level and location of facilities

As expected, costs are higher in higher level, and urban, facilities. The level of facility is significant for both inpatient and outpatient care costs, with p < 0.001 for all levels. With regards to inpatient care, specialist and teaching hospitals (levels 4 and 5) have higher estimated unit cost than district hospitals (level 3). Urban/rural location is significant for outpatient care costs, with higher unit costs in urban settings.

### Size of facilities

The size of facilities providing inpatient care is measured by the number of admissions (used instead of number of beds as the latter was highly collinear with the occupancy rate). This parameter has a very small, but still significant (p < 0.05) positive effect on inpatient costs. The small effect on cost can be said to result from mixed effects because, on the one hand, higher admissions could lead to lower overhead cost per patient and greater efficiency while, on the other hand, greater size could also indicate more specialist care with a larger proportion of complicated cases and thus a higher unit cost. The proper identification of the effect size would require more information of the capacity at which the facility is operating, and more detailed data on resource use at the level of the specialized units within each facility. For outpatient costs, the number of visits per year was used as an indicator of size, and here the results align with our expectations that larger facilities would have lower costs, likely due to operating with more efficient provision of care (significant at p < 0.05).

### Ownership

Compared to facilities run by not for profit private providers (i.e. missions or non-governmental organizations), unit costs are predicted to be lower for both outpatient and inpatient care in public facilities (p < 0.01 in both models). Both models also indicate higher costs in private for profit facilities (highly significant at p < 0.01 for inpatient care, and somewhat less significant at p < 0.05 for outpatient care). These results align with our expectations [22, 23].

### Capacity utilization

The models confirm measures of capacity utilization as important explanatory variables of cost [3]. A higher bed occupancy rate should result in lower inpatient bed day cost, as fixed costs are spread across a greater number of outputs. This effect is confirmed by the inpatient model (p < 0.01). A longer average length of stay (ALOS) is significantly associated with lower inpatient bed day cost (p < 0.001), presumably because fixed costs are spread over a greater number of days. A greater number of visits per provider per day significantly reduces the outpatient cost (p < 0.001), more so than the number of visits per facility.

### Country dummies

Country dummies were included only where their effect was significant, which is the case for both inpatient costs where Brazil observations constitute 58% of the entire sample, and outpatient costs where Brazil makes up as much as 62% of observations included in the final model. For outpatient care we also included a dummy variable for Colombia. All dummies for Brazil and Colombia are highly significant at p < 0.001.

### Comparison with earlier models

The regression model for inpatient care differs from the earlier WHO-CHOICE prediction model in that variables for food and drug costs are not included [8]. On the other hand we included other variables that have been reported in hospital cost function estimation literature, such as the average length of stay [24, 25].

The model for outpatient care is significantly different from the previous WHO-CHOICE models, in that the new model differentiates between levels of care.

### Validation

For internal validation purposes we produced scatter graphs of actual and predicted observations by facility type and examined these closely for every country. As described above, predicted values were based on the 80th percentile of variables used for prediction (Table 3).

**Fig. 1:**
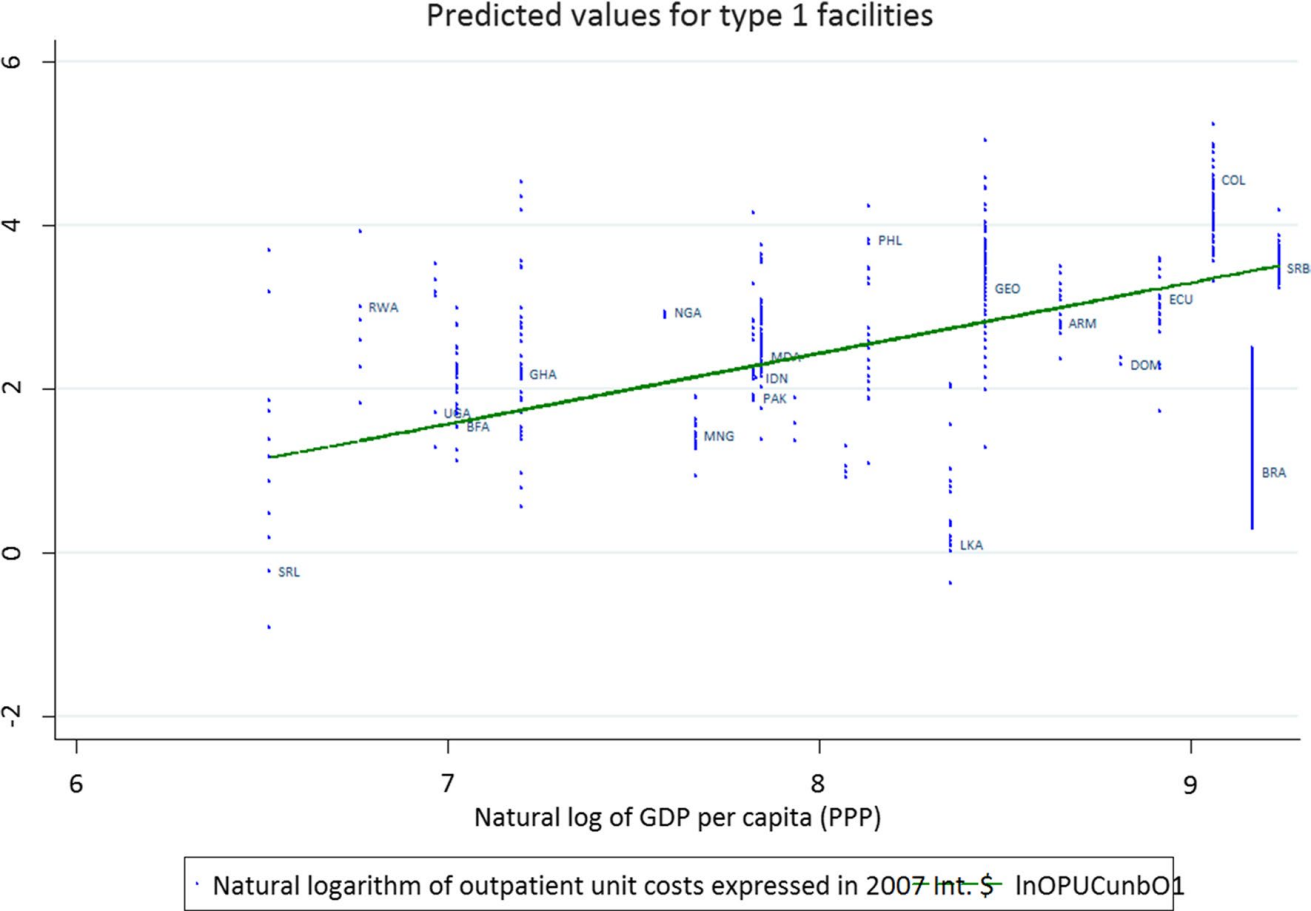
Predicted values (regression lines) for outpatient service delivery costs in level 1 facilities, 2007 I$, plotted against the natural log of GDP per capita (X axis). (Y-axis shows the raw data for cost per visit in natural logs) N = 4750. Natural logarithm of outpatient unit costs expressed in 2007 I$. *ARM* Armenia, *BFA* Burkina Faso, *BRA* Brazil, *COL* Colombia, *GEO* Georgia, *GHA* Ghana, *ECU* Ecuador, *IDN* Indonesia, *MDA* Moldova, *NGA* Nigeria, *MNG* Mongolia, *PAK* Pakistan, *PHL* Phillippines, *RWA* Rwanda, *SRL* Sri Lanka, *USA* USA

Figure 1 plots the predicted values from the outpatient model against the unit cost data and the level of GDP per capita, for all level 1 facilities for the countries included in the sample. The line represents the predicted values of the cost per visit (in natural logs), estimated for a public facility using assumptions outlined in Table 3.

**Fig. 2:**
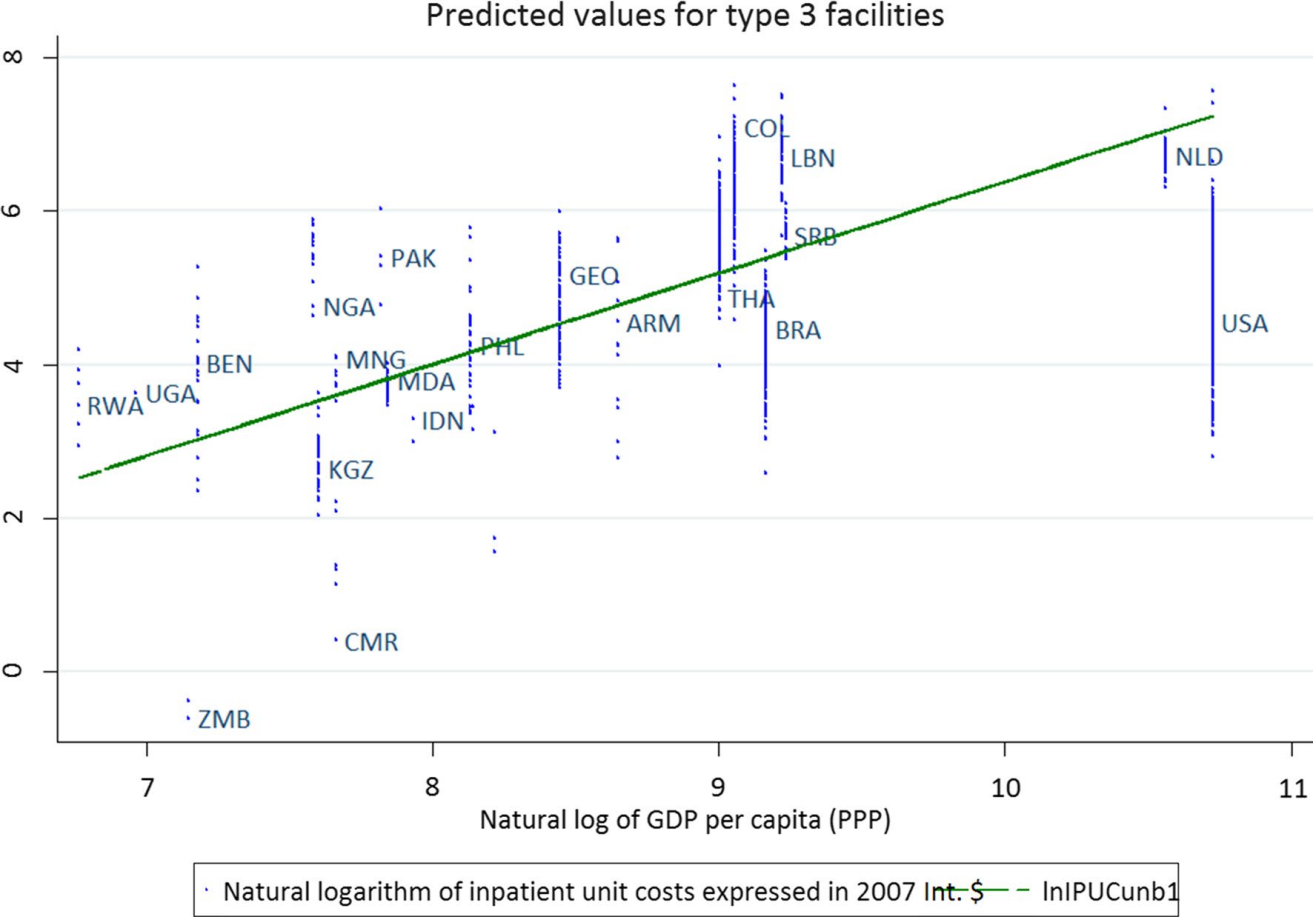
Predicted values (regression lines) for inpatient service delivery costs in level 3 facilities, 2007 I$, plotted against the natural log of GDP per capita (X axis). (Y-axis shows the raw data for cost per inpatient bed day in natural logs) N = 5037. Natural logarithm of inpatient unit costs expressed in 2007 I$. *ARM* Armenia, *BEN* Benin, *BFA* Burkina Faso, *BRA* Brazil, *COL* Colombia, *CMR* Cameroon, *GEO* Georgia, *GHA* Ghana, *ECU* Ecuador, *IDN* Indonesia, *KGZ* Kyrgyzstan, *LBN* Lebanon, *MDA* Moldova, *MNG* Mongolia, *NGA* Nigeria, *NLD* Netherlands, *PAK* Pakistan, *PHL* Philippines, *RWA* Rwanda, *SRL* Sri Lanka, *SRB* Serbia, *THA* Thailand, *UGA* Uganda, *USA* USA, *ZMB* Zambia

Figure 2 similarly plots the predicted values for inpatient service delivery costs against the country supplied estimates and the level of GDP per capita, for level 3 facilities. The predicted costs reflect the regression analysis and as such represent an average relationship between cost determinants, whereas the actual country values demonstrate a significant spread due to facility-specific conditions.

The figures confirm that the models have a reasonable fit with the data and illustrate the considerable variability in the observed unit costs within individual countries (each column of dots represents a country with a specific GDP per capita).

Moreover, we attempted to validate the predicted CHOICE cost estimates against cost data on TB and HIV-related health services (outpatient visits) which had been collected through country-specific research studies by WHO’s TB department and UNAIDS. While methodological differences between studies makes a direct comparison challenging, the comparison indicated that estimates were in the same range.

The models outlined here are used to derive predictions at country and WHO region level. Table 6 presents the predicted values for public hospitals in selected countries using Table 3 assumptions and 95% uncertainty intervals. The estimates are presented in 2010 I$, based on the 2010 GDP per capita in I$. Table 6 and Fig. 2 illustrate the comparatively lower estimates derived from the data samples from Brazil and the United States of America. Additional predictions are available from the WHO-CHOICE website: http://www.who.int/choice.

**Table 6:** Predicted service delivery cost per bed-day (i) for selected countries (2010 I$)

Country (ranked by GDP per capita)	GDP per capita (I$)	Hospital level	Cost per bed day (I$)			
			Mean	95% uncertainty interval low	95% uncertainty interval high	SD
Mozambique	894	3	6.97	2.74	15.29	3.15
		4	7.46	2.81	15.58	3.37
		5	9.76	3.56	21.80	4.62
Mali	1215	3	10.17	4.05	21.49	4.72
		4	10.78	4.21	22.94	4.99
		5	14.40	5.45	30.34	6.59
Indonesia	4298	3	46.61	19.03	102.61	21.68
		4	48.61	19.83	102.41	20.87
		5	62.97	23.33	139.86	28.97
Algeria	8029	3	98.15	104.21	134.41	35.25
		4	104.21	134.41	35.25	38.34
		5	134.41	35.25	38.34	52.49
Ecuador	8795	3	105.79	41.16	215.84	46.98
		4	113.27	44.88	244.39	53.20
		5	151.41	61.03	322.49	68.84
Brazil	11,187	3	27.96	11.31	63.05	12.85
		4	30.18	11.46	63.20	13.44
		5	37.96	14.63	78.69	16.61
Romania	14,815	3	202.22	79.57	439.38	93.24
		4	210.68	88.23	466.93	96.38
		5	272.20	104.03	577.31	123.74
Bahrain	20,339	3	290.75	305.89	400.24	117.36
		4	305.89	400.24	117.36	118.35
		5	400.24	117.36	118.35	153.11
Russian Federation	20,593	3	296.04	106.32	648.53	138.93
		4	313.35	114.96	678.27	154.36
		5	407.34	153.69	925.76	197.33
Greece	27,545	3	437.22	162.5	943.81	209.06
		4	441.67	174.11	960.97	196.38
		5	569.30	226.01	1206.52	260.50
United Kingdom	35,433	3	570.07	217.32	1265.39	267.32
		4	612.29	218.78	1397.20	299.66
		5	767.92	289.87	1664.19	367.70
Canada	39,176	3	646.49	237.17	1461.88	299.35
		4	680.16	266.49	1532.70	317.30
		5	869.98	331.92	1869.47	403.22
United Arab Emirates	42,180	3	719.15	272.51	1625.10	357.52
		4	740.80	294.82	1629.96	338.45
		5	952.57	381.38	2097.89	426.29
United States of America	46,747	3	788.50	293.85	1700.93	381.24
		4	834.13	318.51	1789.65	375.56
		5	1093.51	415.16	2426.98	535.63

(i) Cost per bed day is estimated for public hospitals, excluding the cost of drugs but including costs such as personnel, capital and food costs

For definition of levels of care see Box 1

List of countries for which estimates are reported here is the same as was reported in Adams et al. [8], with the addition of Brazil and United States of America, which represent large shares of the data sample

*SD* standard deviation

We examined our data set for the reported shares of drug costs which were 10.6% for outpatient care (N = 5478 out of 9028) and 5.3% for inpatient care (2688 out of 3407). While these are likely underestimates, we ran a sensitivity analysis using these shares, with results reported in Table 7.

**Table 7:** Predicted service delivery cost per bed-day and outpatient visit for selected countries, using different ratios for drug cost adjustment

Country	GDP per capita (I$)	Facility level	Cost per outpatient visit				Cost per inpatient bed day			
			Mean (with standard drug % share)^a^		Mean (with lower drug % share)^b^		Mean (with standard drug % share)^a^		Mean (with lower drug % share)^b^	
			2010 I$	2010 USD	2010 I$	2010 USD	2010 I$	2010 USD	2010 I$	2010 USD
Mozambique	894	1	2.2	1.0	3.7	1.7				
	–	2	2.7	1.2	4.5	2.1				
	–	3	3.1	1.4	5.2	2.4	7.0	3.2	12.9	6.0
	–	4	3.2	1.5	5.4	2.5	7.5	3.5	13.5	6.3
	–	5	3.2	1.5	5.4	2.5	9.8	4.6	17.4	8.1
Mali	1215	1	4.0	1.5	6.8	2.6				
	–	2	4.8	1.9	8.2	3.2				
	–	3	5.6	2.2	9.5	3.7	10.2	3.9	18.6	7.2
	–	4	5.7	2.2	9.7	3.7	10.8	4.2	19.3	7.5
	–	5	5.8	2.2	9.9	3.8	14.4	5.6	25.0	9.7
Indonesia	4298	1	15.0	5.7	25.6	9.6				
	–	2	18.5	7.0	31.4	11.8				
	–	3	20.7	7.8	35.2	13.3	46.6	17.6	83.1	31.3
	–	4	21.2	8.0	36.1	13.6	48.6	18.3	88.4	33.3
	–	5	20.9	7.9	35.6	13.4	63.0	23.7	113.1	42.6
Algeria	8029	1	21.6	7.6	36.8	13.0				
	–	2	25.0	8.9	42.6	15.1				
	–	3	29.5	10.4	50.3	17.8	98.2	34.7	176.3	62.4
	–	4	30.6	10.8	52.1	18.5	104.2	36.9	186.4	66.0
	–	5	31.5	11.2	53.7	19.0	134.4	47.6	234.2	82.9
Brazil	8795	1	4.8	3.8	8.2	6.5				
	–	2	5.8	4.5	9.8	7.7				
	–	3	5.4	4.2	9.2	7.2	28.0	22.0	51.2	40.3
	–	4	7.0	5.5	12.0	9.4	30.2	23.8	52.7	41.6
	–	5	6.9	5.5	11.8	9.3	38.0	29.9	67.2	52.9
Romania	14,815	1	27.9	13.5	47.5	22.9				
	–	2	33.8	16.3	57.5	27.8				
	–	3	37.5	18.1	63.8	30.8	202.2	97.7	371.4	179.4
	–	4	40.6	19.6	69.2	33.4	210.7	101.7	384.1	185.5
	–	5	41.1	19.8	69.9	33.8	272.2	131.5	493.0	238.1
Russian Federation	20,593	1	31.9	16.6	54.3	28.3				
	–	2	38.8	20.2	66.0	34.4				
	–	3	42.4	22.1	72.3	37.6	296.0	154.2	537.4	279.9
	–	4	46.2	24.1	78.7	41.0	313.4	163.2	561.3	292.3
	–	5	47.3	24.7	80.6	42.0	407.3	212.1	741.0	385.9
United Arab Emirates	56,415	1	77.1	47.9	131.3	81.6				
	–	2	95.9	59.6	163.3	101.5				
	–	3	109.5	68.0	186.4	115.8	719.2	446.8	1275.1	792.2
	–	4	108.8	67.6	185.2	115.1	740.8	460.2	1299.2	807.2
	–	5	112.6	70.0	191.8	119.2	952.6	591.8	1726.3	1072.5

Costs are estimated per bed day for public health facilities, excluding the cost of drugs. ^a^ Standard drug share uses 47.5% assumption for medicines ^b^ Lower drug shares were derived from the sample as 10.6% for outpatient care and 5.3% for inpatient care. For definition of levels of care see Box 1. Cost estimates were transformed from I$ to US$ using exchange rates for 2010 available from World Development Indicators (Accessed 19 September 2017)

## Discussion

This paper describes the most recent effort by WHO to develop models to predict country-specific costs for outpatient visits and inpatient bed days. The database of country-specific cost estimates is a public good provided by WHO which serves a unique purpose at global, regional, and country level, allowing analysts to easily access data and apply these within a range of analytical settings—including economic evaluation, cost of illness studies, investment cases and resource needs appraisals.

The models presented in this paper were informed by data gathered through a thorough search for databases that reported costs at a range of facilities. Data imputation techniques were explored but not used, given that they reduced the performance of the models according to statistical and validation tests when compared to models not including imputed values. This probably indicates that data sets with missing values are less reliable than other data, which further suggests that a ‘missing at random’ assumption is inappropriate for this sample.

The variables included in the models have high statistical significance and the signs of the coefficients are consistent with results from previously published models. While there is a significant variation at country level (Figs. 1, 2), the model clarifies how such variation may be due to type/level of facility, facility size, ownership, and current capacity utilization. While a substantial portion of the observed variability can be explained by the specified determinants, some unexplained variability remained, possibly due to factors that we could not measure such as case mix (variation in diagnosis), quality of care, and incentive structures.

The majority of data points that the model draws upon refer to year 2007. While technology may have since evolved, the revised WHO CHOICE estimates presented here provide a valuable resource and more recent estimates compared to previous analysis. It is recommended that new rounds of data collection be conducted in the future to guide further model updates. In the interim however, the current model provides researchers and analysts with a set of comparative cost estimates not found elsewhere. Moreover, additional future work will be needed to improve cost estimates for outreach and community service delivery platforms which are important in particular for prevention activities.

### Comparison with estimates derived from previous WHO-CHOICE prediction models

As expected, when compared with the previous round of WHO-CHOICE models, [3, 8, 9] the models presented here result in predicted costs that are higher in most cases, particularly for outpatient care. The background technical report provides more information on comparative statistics between the past and current set of models [26]. Higher costs from the current set of models are expected due to changes in technology as well as general price inflation.

The updated estimates are based on data from fewer countries than the previous WHO-CHOICE regression analysis (30 compared to 80). However, when considering the considerable variation in unit costs reported within countries the recommendation is that a sufficiently large number of facilities within a country is required to ensure representativeness [9]. Therefore, if one considers only datasets with 10 or more observations, the number of datasets in the two analyses are similar (30 in the new analysis compared to 33 in the first round) and can thus be considered similarly representative of between-country variation. With a higher average number of facilities per country, the new dataset is more representative of within-country variation. A significant limitation in the new round of analysis is however the lack of outpatient cost data from high-income countries. The effect of this feature of the data on the comparability of cost estimates across both hospitals and health centers is unknown.

### Caveats

Unit cost estimates are sensitive to the method used for cost allocation [27, 28]. Most data collectors reported using a bottom up approach (53% of sample, with 14% using a top-down approach and 33% not providing information, data not shown). The list of variables collected varied across settings, which is expected as costs would have been collected for different purposes. Nevertheless this posed challenges for our aggregate analysis. A particular challenge concerned overhead costs, where analysts included different components, which makes it likely that some respondents underestimated overhead costs. Another challenge was health worker salaries, where analysts reported encountering difficulties locating relevant cost data, particularly in public sector settings where managers have limited information on salaries of their fellow co-workers. A few respondents reported allocation between inpatient and outpatient care based on revenue generation rather than resource use. Estimates were pooled even when revenue generation was reported.

Data providers should be better equipped to extract commodity costs from their estimates. The use of a uniform ratio across countries to remove food- and/or drug related costs is a limitation, as the ratio of drugs relative to other costs would presumably vary across settings [29, 30]. Moreover, the average contribution of drug costs derived from previous work (47.5%) is higher than expected. The World Health Report 2010 reported that pharmaceuticals account for 20–30% of all global health spending [1]. Nevertheless, the 47.5% assumption is retained for this round of CHOICE estimates. Table 7 provides a sensitivity analysis highlighting the impact of this assumption.

Similarly, measures around performance of health facilities and overall capacity should be incorporated during cost data collection processes. The above challenges are a result of using secondary data sources but nevertheless point to lack of standardised data collection and reporting processes.

### User-defined parameters

Our choice of admissions as the size measure for hospitals may be critiqued in that this reflects short-run output volume variations while hospital beds would better reflect installed capacity [31].

Moreover, in order to derive a standard set of WHO-CHOICE cost estimates (Table 4) we applied specific assumptions derived from the data sample, for facilities operating at the 80% percentile of a sample of similar such facilities in terms of capacity utilization and output. It is possible the 80% percentile values do not correspond to performance at 80% capacity, but unfortunately the data does not allow for verification of this. On the assumption that many facilities in low and middle income countries operate at low capacity, our prediction values may be too low, which would overestimate costs for a system operating at 80% capacity. A spreadsheet model is available through the WHO-CHOICE website for those who wish to adjust the parameters used and generate their own country specific estimates (http://who.int/choice/cost-effectiveness/inputs/en/), for example in relation to facilities that are private for profit, or facilities located in rural areas. Different assumptions for the predictor variables (e.g. average length of stay, number of patients seen per provider per day, etc.) may be used, although it is recommended that unit costs for economic evaluations should reflect technically and economically efficient service provision [32].

## Conclusions

WHO-CHOICE health service delivery unit costs are unique and considered a standard data source for economic analysis, used by analysts all over the world. With the new models, unit cost estimates have been produced for all 14 WHO epidemiological sub-regions (and 21 regions used by the IHME for Global Burden of Disease Project) [33], using population-weighted GDP per capita. The models can predict country-specific unit costs at different capacity levels and in different settings. Country and region estimates are available through the WHO-CHOICE website, along with tools that allow users to adjust variables and make their own predictions [34].

With the SDGs there is a call to action to maximise the efficiency of data collection, and for informed and accountable decision making [35]. Resource allocation should be based on informed analysis with regards to costs and benefits. It is therefore critically important that national and local governments as well as the international community strengthen efforts to collect, analyse, and make use of information on health system resource use and efficiency. Efforts should be made to integrate cost information in general data collection efforts. For example, the World Bank’s Service Delivery Indicators program has been collecting facility-level data on performance and quality of service delivery indicators for a limited number of countries in Africa (Kenya, Senegal, Tanzania and Uganda) but to our knowledge has not focused on service costs [36]. Similarly with the District Health Information Software (DHIS2) software platform being used in a large number of countries, there is now ongoing work to assess how information on facility level resource use can be regularly captured and used to inform decision making. Countries should develop systematic data collection systems to store, transfer and produce robust and up to date strategic financial information for stakeholders at local, sub-national and national levels [2].

There is a need to strengthen capacity to understand and make use of data at country level, in particular in low-income countries where resources are limited and the use of economic and financial data for evaluating current system performance could lead to considerable efficiency gains [1]. The WHO-CHOICE project makes tools, models and datasets available for county users and has recently invested in the enhanced user-friendliness of the suite of cost-effectiveness tools through their incorporation into the Spectrum platform, and a built-in link to the OneHealth Tool—a joint UN resource projection tool used in over 40 countries [37].

While the WHO-CHOICE project provides robust defaults for countries to assess costs at five levels of service delivery, future modelling work should consider additional delivery modes such as community based delivery and outreach. More research may be needed regarding the assumptions used for continuous independent predictor variables in order to derive country predicted values, and guidance regarding setting norms for capacity utilization.

## Additional file

**Additional file 1: Annex S1.** Countries and number of unit cost observations included in the inpatient and outpatient cost prediction models. **Annex S2.** Data characteristics collected through standard template.
